# Stochastic integrated model-based protocol for volume-controlled ventilation setting

**DOI:** 10.1186/s12938-022-00981-0

**Published:** 2022-02-11

**Authors:** Jay Wing Wai Lee, Yeong Shiong Chiew, Xin Wang, Mohd Basri Mat Nor, J. Geoffrey Chase, Thomas Desaive

**Affiliations:** 1 School of Engineering, Monash University Malaysia, Subang Jaya, Selangor Malaysia; 2 Kulliyah of Medicine, International Islamic University Malaysia, Kuantan, Malaysia; 3 Center of Bioengineering, University of Canterbury, Christchurch, New Zealand; 4 GIGA In-Silico Medicine, University of Liege, Liege, Belgium

**Keywords:** Mechanical ventilation, Stochastic modelling, Respiratory mechanics, Decision-making, Model-based protocol, Critical care

## Abstract

**Background and objective:**

Mechanical ventilation (MV) is the primary form of care for respiratory failure patients. MV settings are based on general clinical guidelines, intuition, and experience. This approach is not patient-specific and patients may thus experience suboptimal, potentially harmful MV care. This study presents the Stochastic integrated VENT (SiVENT) protocol which combines model-based approaches of the VENT protocol from previous works, with stochastic modelling to take the variation of patient respiratory elastance over time into consideration.

**Methods:**

A stochastic model of *E*_rs_ is integrated into the VENT protocol from previous works to develop the SiVENT protocol, to account for both intra- and inter-patient variability. A cohort of 20 virtual MV patients based on retrospective patient data are used to validate the performance of this method for volume-controlled (VC) ventilation. A performance evaluation was conducted where the SiVENT and VENT protocols were implemented in 1080 instances each to compare the two protocols and evaluate the difference in reduction of possible MV settings achieved by each.

**Results:**

From an initial number of 189,000 possible MV setting combinations, the VENT protocol reduced this number to a median of 10,612, achieving a reduction of 94.4% across the cohort. With the integration of the stochastic model component, the SiVENT protocol reduced this number from 189,000 to a median of 9329, achieving a reduction of 95.1% across the cohort. The SiVENT protocol reduces the number of possible combinations provided to the user by more than 1000 combinations as compared to the VENT protocol.

**Conclusions:**

Adding a stochastic model component into a model-based approach to selecting MV settings improves the ability of a decision support system to recommend patient-specific MV settings. It specifically considers inter- and intra-patient variability in respiratory elastance and eliminates potentially harmful settings based on clinically recommended pressure thresholds. Clinical input and local protocols can further reduce the number of safe setting combinations. The results for the SiVENT protocol justify further investigation of its prediction accuracy and clinical validation trials.

## Background

Mechanical ventilation (MV) is the primary form of care given to patients with respiratory failure to maintain adequate blood oxygenation and removal of carbon dioxide [1]. Early mechanical ventilators such as the iron lung [2] used negative-pressure ventilation, which used pressure gradients to move air into the patient’s lungs by creating a sub-atmospheric pressure around the patient chest, which required patients to be placed in metal tanks. However, negative-pressure ventilation was cumbersome, restricted both patient motion and the view needed for clinicians to observe patient condition, and required a large amount of space for a single unit. Consequently, virtually all modern ventilators use positive-pressure ventilation, where positive airway pressure is delivered to patient lungs either invasively via intubation or non-invasively via sealed masks covering the face. They are also able to support patient breathing through a large range of pressure and volume control modes [3]. While positive-pressure ventilation removes the obstacles faced by negative-pressure, it also introduces risks, such as barotrauma and volutrauma [4, 5], especially at suboptimal ventilation settings. Hence, safely selecting effective MV settings remains a challenge. Provided with an overwhelming number of possible combinations of settings, clinicians choose based on general guidelines, personal experience, and intuition [6–9], leading to significant variation in care and outcomes.

While the current standard of care is based on years of research and landmark trials [4, 5, 10–13], current guidelines are general and follow a ‘*one-size-fits-all*’ approach, which cannot account for inter- or intra-patient variability [14–16]. Non-patient-specific guidelines benefit some patients, but also yield suboptimal care for others [17]. Consequently, patient response ultimately guides changes in care, leading to a trial-and-error approach [18], meaning patients may experience suboptimal MV settings. Thus, suboptimal MV is not uncommon and can cause ventilator-induced lung injury, increasing the risk of negative outcomes [19, 20].

The next step in improving care is individualised treatment based on patient-specific response. While many tools, such as computed tomography (CT) scans [21], lung ultrasound [22] and electrical impedance tomography [23], exist to detect and measure lung recruitability, they are rarely employed due to lack of resources or time, difficulty, and added clinical burden [24]. However, model-based methods provide non-invasive and non-intrusive alternatives, by using readily available breath data from patients to identify patient-specific parameters [16, 24, 25]. In the context of respiratory mechanics, these patient-specific parameters are known as respiratory elastance (*E*_rs_) and respiratory resistance (*R*_rs_), which represents the elastic properties of the respiratory system and the resistance to air flow of the respiratory system, respectively. Elastance is the rate at which pressure results in volume recruited, while resistance is related to the pressure required to overcome resistance to flow. The two components result in the total pressure required to deliver a given volume in a given time to a patient. Clinically, *E*_rs_ is more familiar in its reciprocal, respiratory compliance. Many studies have recommended MV settings based on *E*_rs_ or a surrogate [26–34].

Hence there is a potential role for computerised automatic systems for continuous monitoring and selection of MV settings to improve individualised patient care and reduce clinical burden. There are several successful works in this area, including but not limited to PRICO (Acutronic, Hirzel, Switzerland) [35] and CLiO_2_ (Vyaire Medical, Mettawa, USA) [36]. These are examples of automated oxygen control [37] that automate FiO_2_ input settings for neonatal patients using differential feedback and rule-based control based on current SpO_2_ information, to keep SpO_2_ levels within target ranges. Other works include but are not limited to IntelliVENT-ASV (Hamilton Medical, Bonaduz, Switzerland) [38], and Beacon Caresystem (Mermaid Care A/S, Nørresundby, Denmark) [39, 40]. These latter two examples combine clinical rules together with physiological models to provide recommendations for other MV settings such as tidal volume, respiratory rate, and minute ventilation. However, given recent discoveries regarding the importance of respiratory elastance and driving pressure and its implications on patient outcome [31], there is potential to further improve these decision support systems based on other physiological models.

However, *E*_rs_ varies with both time and patient condition, as well as changes in MV settings [41]. As such, capturing the variability of *E*_rs_ over time may prove essential to enable the selection of safe patient-specific MV settings at any given time, where safe is defined as when key parameters are within literature recommended safety thresholds. Clinically, stochastic modelling has demonstrated its clinical utility and impact in managing variability in glycemic control in the intensive care unit (ICU) [42, 43].

In previous works [44], a preliminary stochastic model of *E*_rs_ was over time was developed. The result was a stochastic model of *E*_rs_ with promising cross-validation results of 92.59% and 68.56% of forecasted *E*_rs_ within the 5–95% and 25–75% prediction range, respectively. In a separate work [45, 46], a decision support system protocol known as the ‘VENT’ protocol was developed, which utilises a physiological lung model together with well-established clinical rules to provide a narrowed range of recommended MV settings for clinicians. The previous VENT protocol showed potential in reducing an overwhelming number of possible MV settings to a safe narrowed range of settings that were all deemed safe according to literature recommendations of pressure and volume outcomes. However, the VENT protocol assumes that patient-specific *E*_rs_ remains constant for an approximate period of time and cannot take the variation of *E*_rs_ over time into consideration. Hence, this paper aims to assimilate the two works to form the stochastic integrated VENT (SiVENT) protocol.

This research integrates stochastic modelling with model-based methods to assist clinicians in selecting safe patient-specific MV settings, directly accounting for inter- and intra-patient variability. It also assesses if stochastic modelling adds benefit to model-based care approaches. A well-validated respiratory model is used with patient data and incorporated with a stochastic model of *E*_rs_ to simulate combinations of ventilation settings, which are tested in a performance evaluation study to assess the MV decision support protocol.

## Results

**Fig. 1:**
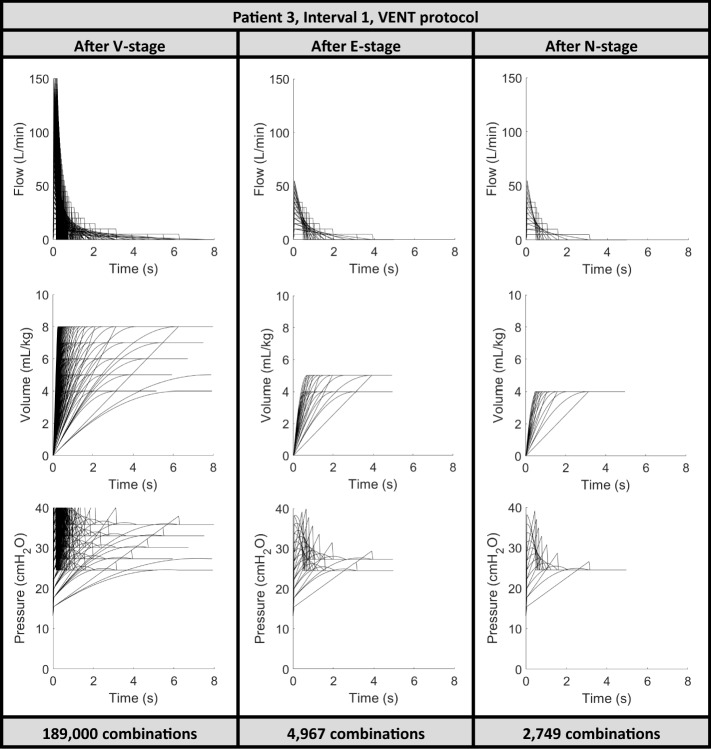
Reduction of MV setting combinations for Patient 3, interval 1 in each stage of the VENT protocol [45, 46], where the *V*-stage refers to the ‘Virtually Ventilate’ stage, *E*-stage refers to the ‘Eliminate/Estimate’ stage and the N-stage refers to the ‘Narrowing Objectives’ stage

**Fig. 2:**
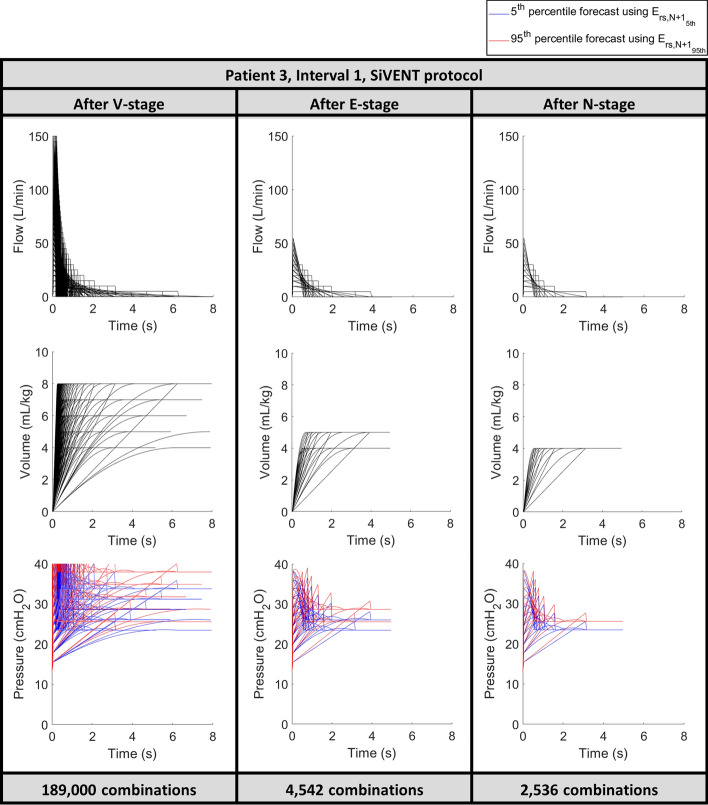
Reduction of MV setting combinations for Patient 3, interval 1 in each stage of the SiVENT protocol, where the* V*-stage refers to the ‘Virtually Ventilate’ stage, E-stage refers to the ‘Eliminate/Estimate’ stage and the *N*-stage refers to the ‘Narrowing Objectives’ stage

Table 1 shows the performance evaluation results for all 20 patients. Patients 10–13 have been omitted due to having less than 3 h of patient data within a single day of ventilation [44]. The initial number of available MV settings in VC ventilation is 189,000. With implementation over the course of 3 h, the VENT and SiVENT protocols have reduced this initial number of settings to a median of 10,612 and 9329 setting combinations, respectively, achieving a median percentage reduction of setting combinations of 94.4% and 95.1%, respectively. Table 2 shows a breakdown of recommendations provided by each protocol at every 10-min interval based on *E*_rs*,N*_ and *R*_rs*,N*_ for Patient 3. Figure 1 illustrates how the number of MV setting combinations is reduced in each stage of the protocol for Patient 3, interval 1 for the VENT protocol. Figure 2 shows the same for the SiVENT protocol, but with the addition of blue and red lines representing the forecast pressure waveforms for the 5th percentile and 95th percentiles, respectively.

**Table 1:** Performance evaluation results comparison between VENT and SiVENT protocol

Patient no.	Weight (kg)	*E*_rs*,N*_ [IQR] (cmH_2_O/L)	*R*_rs*,N*_ [IQR] (cmH_2_O.s/L)	PEEP_*N*_ [IQR] (cmH_2_O)	No. of settings after protocol [IQR]		Percentage reduction in settings [IQR] (%)	
					VENT	SiVENT	VENT	SiVENT
1	52.0	26.0 [25.1–28.9]	7.2 [7.0–7.8]	3 [2–3]	11,179 [11,179–11,179]	11,179 [11,179–11,179]	94.1 [94.1–94.1]	94.1 [94.1–94.1]
2	70.2	72.8 [72.4–73.6]	2.2 [2.1–2.2]	7 [7–7]	10,999 [10,999–10,999]	0 [0–0]	94.2 [94.2–94.2]	100 [100–100]
3	65.0	47.1 [43.7–49.8]	19.3 [13.8–25.6]	13 [13–13]	4306 [2960–6012]	4203 [2749–5811]	97.7 [96.8–98.4]	97.8 [96.9–98.5]
4	81.0	25.7 [25.5–26.5]	12.1 [11.9–13.6]	10 [10–10]	8947 [7959–9140]	8554 [7763–8753]	95.3 [95.2–95.8]	95.5 [95.4–95.9]
5	38.0	38.7 [38.5–39.9]	4.5 [4.3–5.3]	7 [7–7]	11,238 [11,238–11,238]	11,238 [11,238–11,238]	94.1 [94.1–94.1]	94.1 [94.1–94.1]
6	70.2	8.1 [7.5–10.2]	18.0 [17.0–18.6]	7 [7–7]	7302 [6910–7694]	7109 [6716–7502]	96.1 [95.9–96.3]	96.2 [96.0–96.4]
7	44.2	33.6 [30.7–35.6]	9.1 [8.8–9.4]	9 [9–9]	11,222 [11,222–11,222]	11,222 [11,222–11,222]	94.1 [94.1–94.1]	94.1 [94.1–94.1]
8	79.4	33.4 [32.1–35.6]	13.2 [12.8–13.8]	10 [10–10]	7587 [7188–7985]	7388 [6990–7788]	96.0 [95.8–96.2]	96.1 [95.9–96.3]
9	53.7	29.3 [27.3–32.0]	4.2 [3.6–7.7]	8 [8–8]	11,156 [11,156–11,156]	11,156 [11,156–11,156]	94.1 [94.1–94.1]	94.1 [94.1–94.1]
14	54.0	13.0 [12.5–13.9]	8.2 [7.3–8.5]	10 [10–10]	11,148 [11,148–11,148]	11,148 [11,148–11,148]	94.1 [94.1–94.1]	94.1 [94.1–94.1]
15	75.0	40.8 [39.5–43.0]	6.6 [6.2–9.1]	11 [11–11]	10,993 [10,034–10,993]	10,993 [9643–10,993]	94.2 [94.2–94.7]	94.2 [94.2–94.9]
16	65.0	36.5 [36.1–38.9]	10.2 [8.1–11.9]	12 [12–12]	9620 [8364–11,056]	9235 [8171–10,865]	94.9 [94.2–95.6]	95.1 [94.3–95.7]
17	80.0	37.8 [35.7–38.5]	10.3 [9.1–13.0]	7 [7–8]	10,128 [8177–10,899]	9741 [7980–10,515]	94.6 [94.2–95.7]	94.8 [94.4–95.8]
18	97.3	38.0 [37.0–39.4]	12.3 [11.4–13.3]	12 [12–12]	6490 [5875–7102]	6081 [5464–6490]	96.6 [96.2–96.9]	96.8 [96.6–97.1]
19	56.0	28.5 [27.6–28.8]	10.0 [9.8–10.2]	10 [10–10]	11,154 [10,966–11,154]	10,966 [10,777–11,154]	94.1 [94.1–94.2]	94.2 [94.1–94.3]
20	72.0	47.1 [46.9–47.7]	12.1 [11.9–12.5]	10 [10–10]	8092 [7498–8092]	7597 [7102–7896]	95.7 [95.7–96.0]	96.0 [95.8–96.2]
21	50.0	58.3 [56.8–61.4]	17.9 [13.4–18.2]	10 [10–10]	5652 [5458–7791]	5458 [5458–7791]	97.0 [95.9–97.1]	97.1 [95.9–97.1]
22	91.9	32.7 [32.0–33.4]	7.2 [6.9–8.4]	12 [12–12]	10,744 [9779–10,744]	10,260 [9390–10,550]	94.3 [94.3–94.8]	94.6 [94.4–95.0]
23	60.0	20.0 [19.1–22.5]	15.1 [14.9–15.5]	14 [14–14]	6463 [6270–6463]	6270 [6076–6270]	96.6 [96.6–96.7]	96.7 [96.7–96.8]
24	62.0	12.7 [12.5–13.0]	5.3 [5.2–6.5]	9 [9–9]	11,097 [11,097–11,097]	11,097 [11,097–11,097]	94.1 [94.1–94.1]	94.1 [94.1–94.1]
Median [IQR]	65 [53.9–77.2]	33.8 [25.6–40.1]	9.8 [6.9–13.3]	10 [8–11]	10,612 [7587–11,148]	9329 [6754–11,148]	94.4 [94.1–96.0]	95.1 [94.1–96.4]

**Table 2:** Patient 3 interval breakdown for performance evaluation results comparison between VENT and SiVENT protocol

Interval breakdown for patient 3							
Interval, *N*	*E*_rs*,N*_ (cmH_2_O/L)	*R*_rs*,N*_ (cmH_2_O.s/L)	PEEP_*N*_ (cmH_2_O)	No. of settings after protocol		Percentage reduction in settings (%)	
				VENT	SiVENT	VENT	SiVENT
1	43.5	26.2	13	2749	2536	98.5	98.7
2	43.7	26.6	13	2749	2536	98.5	98.7
3	44.1	26.8	13	2749	2536	98.5	98.7
4	43.5	25.6	13	2960	2749	98.4	98.5
5	46.7	23.8	13	2960	2960	98.4	98.4
6	43.8	26.8	13	2749	2536	98.5	98.7
7	51.0	20.7	13	3585	3378	98.1	98.2
8	53.9	20.1	13	3585	3378	98.1	98.2
9	42.2	21.1	2	6404	6208	96.6	96.7
10	48.7	14.3	13	5610	5610	97.0	97.0
11	50.1	14.2	13	5610	5412	97.0	97.1
12	49.7	13.8	13	6012	5811	96.8	96.9
13	49.8	13.2	13	6211	6211	96.7	96.7
14	53.3	13.8	13	5811	5613	96.9	97.0
15	47.5	13.0	13	6409	6211	96.6	96.7
16	43.7	13.6	13	6210	6012	96.7	96.8
17	49.5	18.3	13	4203	4203	97.8	97.8
18	44.9	18.5	13	4408	4203	97.7	97.8
Median [IQR]	47.1 [43.7–49.8]	19.3 [13.8–25.6]	13 [13–13]	4306 [2960–6012]	4203 [2749–5811]	97.7 [96.8–98.4]	97.8 [96.9–98.5]

## Discussion

The parameter of interest is *E*_rs_, a patient-specific parameter reflecting the elastic properties of the respiratory system. A high *E*_rs_ value indicates a stiffer, less compliant lung, requiring higher pressures and work to inflate the lungs. As such, some methods of selecting safe MV settings rely on the monitoring *E*_rs_ to understand the progression of patient condition. For example, one method of setting PEEP relies on using the static pressure–volume curve, where PEEP is set at the point of linear compliance [47] and was associated with improved survival [48, 49]. Another example of setting PEEP is shown in the work of Chiew et al., 2015 [29] where they demonstrated a proof-of-concept of using patient-specific *E*_rs_ to titrate optimal PEEP by setting PEEP to the point of minimum *E*_rs_ which describes the point of minimum stiffness and therefore maximum recruitablity. More recently, Goligher et al. [31] showed low tidal ventilation strategies resulted in significantly different mortality benefits based on patient-specific elastance, similar to *E*_rs_, indicating a clear potential to optimise MV setting selection based on *E*_rs_. However, patient-specific *E*_rs_ varies with time [41]. Hence, stochastic modelling offers a way to capture the variability of *E*_rs_ over time, while following a minimum driving pressure protocol similar to the approach in Goligher et al. [31].

A stochastic model accounting for inter- and intra-patient variability is incorporated into the VENT protocol to form the SiVENT protocol. The largest technical effect of this integration on the model-based protocol used is a doubling of computation, as each possible setting combination is forward simulated twice to forecast pressure waveforms for the 5th and 95th percentile *E*_rs*,N*+*1*_ values from the stochastic model, as illustrated in the blue and red lines in Fig. 2. In comparison, the VENT protocol without the stochastic model generates only one forecast outcome pressure, as shown in Fig. 1.

Clinically, more setting combinations are eliminated in the SiVENT protocol as the range of forecast pressure outcomes must be within clinically accepted safety thresholds. In this way, the stochastic model-integrated protocol takes the variation of *E*_rs_ over time into consideration when recommending MV settings, thus reducing the total number of possibilities by 12% in the median case. Hence, the SiVENT protocol recommends a smaller number of MV setting combinations, all of which meet clinically accepted guidelines for safety and clinically specified goals, in this case to minimise driving pressure, *∆P*.

**Fig. 3:**
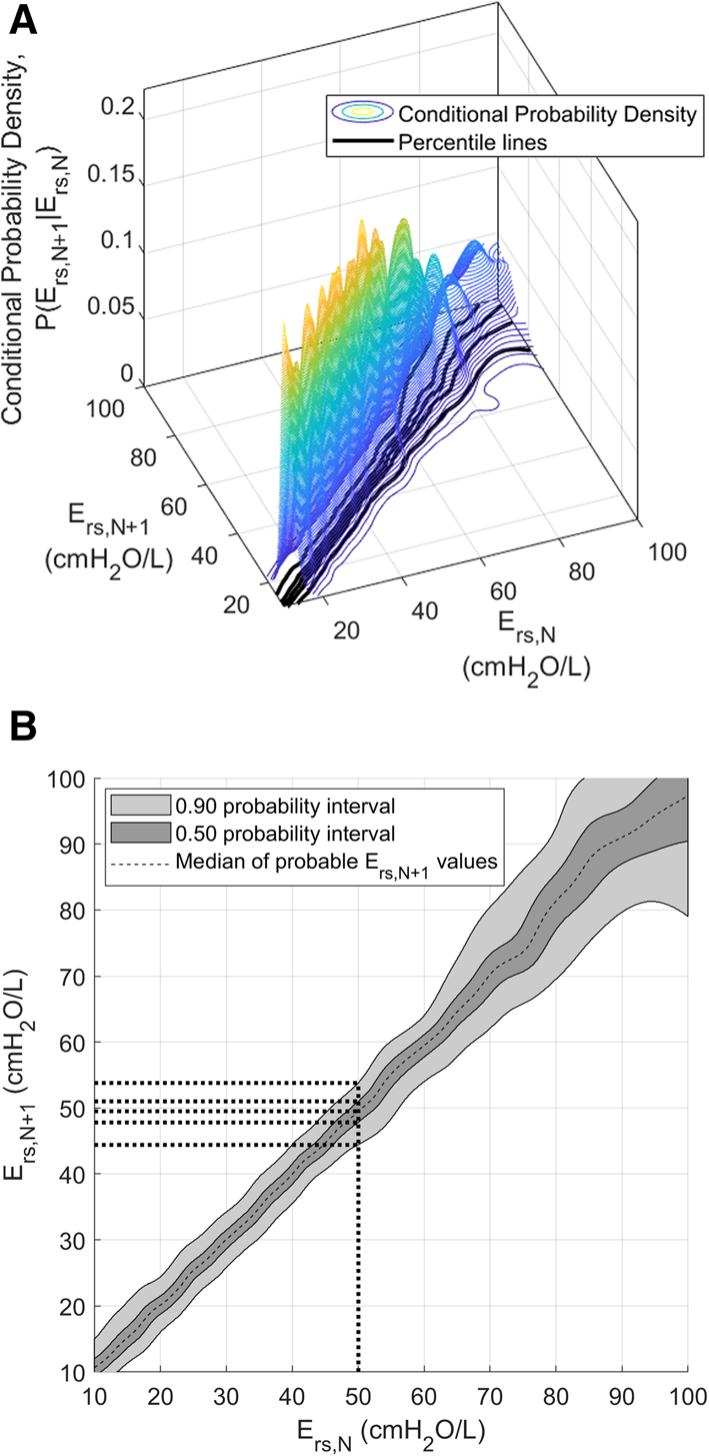
Stochastic model of *E*_rs_ developed by Lee et al. [44] **A** Stochastic model of *E*_*rs*_ in 3-D view. **B** Top view of **A**, showing a more readable 2-D format of the stochastic model. The bold dotted lines illustrate how this can be used as a look-up table to forecast a potential range of *E*_rs*,N+1*_ given *E*_rs*,N*_

However, Patients 1, 5, 7, 14 and 24 experience no difference in number of recommendations provided by VENT or SiVENT. This outcome indicates the SiVENT protocol has determined the variation of *E*_rs_ for these patients will not be large enough to cause any of the MV combination settings to potentially exceed threshold values. In contrast, Patient 2 shows the SiVENT protocol eliminated all 10,999 possible setting combinations found by the VENT protocol, showing how accounting for the variation of *E*_rs_ over time could make all possible settings unsuitable according to clinically accepted guidelines. This outcome typically occurs with much higher values of *E*_rs_ where the 5–95 percentile range of *E*_rs*,N*+*1*_ is wider in Fig. 3, indicating larger variation. Patient 2 had the highest *E*_rs*,N*_ values, which were 25% higher than the second highest patient. In such a case, further clinical decisions need to be considered. This result also highlights how these protocols are simply decision support systems based on model-based approaches and will not be able to replace, but only aid the clinician in the ICU setting.

The stochastic model component improves the E-stage of the VENT protocol by eliminating more setting combinations based on the variation of *E*_rs_. It does not play any role in removing more setting combinations in subsequent stages. As such, it is also observed the remaining number of recommendations by SiVENT have an inter-quartile range of 6754 to 11,148, which is still an immense number of combinations for clinicians to choose from. However, the N-stage in this study uses only one narrowing objective of minimising *∆P*. Further criteria in this stage would be clinically typical, mitigating this issue.

In particular, with only one criterion in the N-stage, both the VENT and SiVENT protocols aggressively reduce the number of recommendations from the E-stage by more than half in almost all patients. This result is highlighted in Figs. 1 and 2 when all combination settings with tidal volumes above 4 mL/kg are eliminated due to the objective of minimising *∆P*. Hence, the large number of recommendations at the end of the protocol can be further reduced by adding more narrowing objectives, such as setting a target range for minute ventilation and/or respiratory rate, a tighter range of tidal volumes, setting a lower threshold for pressure outcomes, minimising plateau pressure, or any other clinically set target.

When looking at the interval breakdown in Table 2, there is rarely significant variation in E_rs,N_ over the course of a single interval. More significant changes occur over longer intervals, such as over 1 h from 43.5 cmH_2_O/L in interval 1, to 51.0 cmH_2_O/L in interval 7. As a result, recommendations by either protocol can stay fairly constant in the case studied here, such as observed throughout intervals 1 to 4 or intervals 10 to 13 in Patient 3. If the *E*_rs*,N*_ does not vary much for a patient, the number of recommendations either protocol will provide will not vary in the number of combinations either. This aspect can be seen in Patients 1, 5, 7, 14 and 24 in Table 1. This issue would be mitigated by longer, potentially clinically more realistic intervals, where 10 min demonstrates the concept and would be suitable for closed-loop control, but a longer interval of 1 h would be clinically more realistic for manual ventilator control by clinical staff.

The interval breakdown in Table 2 also highlights the issues with extracting patient settings from patient breath data. In interval 9 of Patient 3, a PEEP setting of 2 cmH_2_O is extracted from the 10 min worth of breath data, while in all other intervals, it is calculated to be 13 cmH_2_O. In reality, it is likely the clinician did not change the PEEP setting so drastically from 13 cmH_2_O to 2 cmH_2_O and then back to 13 cmH_2_O again in the span of 20 min. What is more likely is that during interval 9, the patient experienced excessive patient effort and asynchrony affecting their respiratory waveforms, making it difficult to extract the proper PEEP setting. This error highlights the limitation arising in short intervals from asynchronies, which would impact closed-loop control approaches without added filtering or asynchrony reconstruction (e.g. [50, 51]).

The results indicate a potential for clinical bedside use in future works once validation is more complete. Ideally, the SiVENT protocol is made into a mobile application to be used in a portable electronic device, i.e. a tablet or desktop and is then integrated with a data acquisition system (e.g. [52, 53]) to monitor and collect pressure–flow data. Initial settings at the beginning of ventilation would first be selected by the clinician. Once at least one interval has passed, patient-specific *E*_rs_ and *R*_rs_ can be identified and clinicians can start utilising the SiVENT protocol as a decision support system. First, patient-specific details such as patient weight, current PEEP settings, and *E*_rs_ and *R*_rs_ would be required to be inputted into the application. With this information as input, the SiVENT protocol will simulate through all possible setting combinations. Once the simulation is completed, the application will provide a narrowed selection of safe recommended MV settings, in which a clinician can inspect to provide more insight before choosing a final setting from the list of recommendations. Clinicians should also be able to specify narrowing objectives based on their clinical goals to further narrow down MV settings. Once chosen and implemented, this process can be repeated for every subsequent interval. In this manner, the SiVENT protocol serves its purpose as an open-loop decision support system to provide further insight into the effects of other possible settings.

Only VC ventilation was considered in this study. However, the method is readily extensible to pressure control (PC) ventilation data. The main difference would be criteria on tidal volumes and peak flows to limit volutrauma-based VILI [54]. Thus, the approach presented is generalisable, and would remain equally clinically relevant, which is a central focus of the SiVENT and VENT approaches use of clinically well-accepted guidelines.

The single-compartment model is well-validated clinically [55, 56]. Furthermore, as prior works by the author have utilised this model in the development of the stochastic model of *E*_rs_ [44] and the VENT protocol [45, 46], the same model is used in this study to allow a more direct comparison between the VENT and SiVENT protocol, enabling a better understanding of the impact the stochastic model alone has on the decision support system protocol. Further study could repeat this approach with more descriptive models better capturing lung mechanics, such as those that incorporate basis functions or nonlinear mechanics [30, 34, 57], or including patient-specific effort for assisted breathing modes [58]. However, as noted, the approach is generalisable as long as a deterministic, physiologically relevant model incorporating patient-specific elastance is employed.

One limitation of this study involves the sample size used to evaluate the performance of the VENT and SiVENT protocols. The protocols are implemented every 10 min on each patient, resulting in the protocols being implemented 18 instances (3 h) for each patient, such as shown in Table 2. Hence, this study implements the protocols in 1,080 instances. While retrospective data from 20 separate patients were used in this study, the protocols were only conducted on only 3 h worth of breath data from each patient, adding to a total of 60 h of breath data being used to evaluate the protocols in this cohort. It is still unclear if this sample size is large enough to show statistically significant results in improving patient care. However, future works point towards performing more in-depth in silico studies in longer trial lengths than just 3 h per patient, to determine what the percentage of time pressure and volume outcomes stay within safety thresholds. Finally, this study utilises simulation to demonstrate the concept. To prove clinical efficacy in an ICU setting requires clinical validation trials to ensure the recommendations provided by the SiVENT protocol would be agreeable to professional clinicians, such as those done to develop the Beacon Caresystem (Mermaid Care A/S, Nørresundby, Denmark) [39, 40]. The proof-of-concept results presented justify such initial clinical studies.

The fact that there exists an overwhelming number of possible MV settings makes it difficult to establish safe and optimal settings on a patient-specific level. For example, several landmark studies have established safe ranges of pressure and volume settings because the large number of possible combinations results in such a large possible range of resultant parameters. For example, the ARDSNet landmark trial [10] established lower tidal volumes resulted in lower mortality rates. The higher mortality rates before this significant landmark trial are indeed the fatalities caused by the large combinations available in MV settings. Since then, the possible recommended MV settings have been decreased as the recommended range of tidal volume has been reduced to a narrower range of 4 to 8 mL/kg. The same can be said for other parameters, such as plateau pressures, where before it was established plateau pressures above 30 cmH_2_O [7] increased barotrauma and ventilator-induced lung injury, the fatalities are expected to have been higher without knowledge of this safety threshold. Hence, by discovering new safety thresholds on a patient-specific basis, the long-term goal of this research aims further reduce fatalities. However, assessing this impact is difficult except by this form of extended comparison.

## Conclusions

A stochastic model-based approach to provide patient-specific MV setting recommendations is presented and its impact is assessed in proof-of-concept simulation studies based on clinical data from 20 ICU patients. It uses patient-specific *E*_rs_ to recommend personalised patient-specific MV settings based on clinically accepted guidelines and clinical specifications. The integration of a stochastic model into the VENT protocol from previous works does not add any extra clinical burden to users. With the addition of the stochastic model, the SiVENT protocol can now provide insights into intra-patient *E*_rs_ variability by taking the variation of *E*_rs_ into consideration. Both the proposed VENT and SiVENT protocols require only readily available patient respiratory pressure and flow data and are thus non-invasive and can be performed over any clinically realistic interval.

The results from the performance evaluation show the SiVENT protocol provides a smaller number of recommended MV settings as compared to the VENT protocol, making it more conservative and safer given it accounts for the potential change of *E*_rs_ in the next time interval. All recommended MV settings provided by SiVENT are considered safe based on the current standard in clinical guidelines. Further reductions could be obtained by adding further typical clinical performance requirements. The overall approach is generalisable and readily adapted to any clinical settings and preferences, and can evolve over time as greater knowledge on safe ventilator setting ranges emerges or changes with new clinical studies. The results presented are proof-of-concept, but justify initial clinical studies to assess the impact on clinical workload and patient safety.

## Methods

### Stochastic model of respiratory elastance

Firstly, the stochastic model requires the use of a physiological lung model to identify patient-specific parameters that describe patient condition. While there are a variety of physiological models that can describe lung mechanics in a complex and detailed manner, it is important that the model in question used is easily identifiable, which is of singular importance so it can be used with readily available clinical data. The single-compartment linear lung model is a clinically well-validated physiological model used to describe pulmonary respiratory mechanics [55, 56] and serves as the basis for all other models. The single-compartment model is described below in Eq. 1:

1 $$P_{aw} \left( t \right) = E_{rs} V\left( t \right) + R_{rs} \dot{V}\left( t \right) + P_{0} ,$$

where *P*_*aw*_ represents the airway pressure (cmH_2_O), *t* is time, *V* represents the volume of air delivered to the lungs (L) and *V̇* is the flow of air (L/s). The respiratory elastance (cmH_2_O/L) and respiratory resistance (cmH_2_O.s/L) are represented by *E*_rs_ and *R*_rs_, respectively. *P*_*aw*_ is non-zero when *V* and *V̇* are zero, and thus, *P*_*0*_ is added as offset pressure. This offset pressure is required during MV to keep the lungs from collapsing completely, and thus represents the PEEP applied by a mechanical ventilator if there is little or no intrinsic PEEP [59], yielding:

2 $$P_{aw} \left( t \right) = E_{rs} V\left( t \right) + R_{rs} \dot{V}\left( t \right) + \text{PEEP}.$$

*E*_rs_ is a patient-specific time-varying parameter which is affected by MV settings and evolves with patient condition [41]. Thus, personalised care based on elastance must account for this variability. Stochastic modelling offers a way to capture the variability of *E*_rs_ over time, grouping undefined, diverse variation into a stochastic variable to better describe a dynamic system, increasingly used as a tool to describe complex biological dynamics [60, 61]. It has also found clinical use capturing the evolution of patient-specific insulin sensitivity in a clinical standard of care ICU glycemic control approach [62–66]. This research utilises a stochastic model of *E*_rs_ developed by Lee et al. [44] and is shown in Fig. 3.

The stochastic model was developed using kernel density estimation [67, 68] and makes use of Bayes Theorem, in which conditional probability is defined:

3 $$P\left( {A|B} \right) = \frac{{P\left( {A,B} \right)}}{P\left( B \right)}.$$

In the context of *E*_*rs*_, where A = *E*_rs*,N*+*1*_ and B = *E*_rs*,N*_, the conditional probability function shown in Eq. 3 can be written as:

4 $$P\left( {E_{rs,N + 1} = x|E_{rs,N} = y} \right) = \frac{{P\left( {E_{rs,N + 1} = x, E_{rs,N} = y} \right)}}{{P\left( {E_{rs,N} = x} \right)}},$$

where *N* is a defined time interval. Thus, *E*_rs*,N*_ and *E*_rs*,N*+*1*_ represent the *E*_rs_ of the current interval and the subsequent interval. The probability of *E*_rs*,N*+*1*_ given *E*_rs*,N*_ can then be calculated using kernel density estimation:

5 $$P\left( {E_{rs,N + 1} = x|E_{rs,N} = y} \right) = \frac{{\mathop \sum \nolimits_{i - 1}^{n} \left( {\frac{{\phi \left( {x;x_{i} , \sigma^{2}_{{x_{i} }} } \right)}}{{p_{{x_{i} }} }}} \right)\left( {\frac{{\phi \left( {y;y_{i} , \sigma^{2}_{{y_{i} }} } \right)}}{{p_{{y_{i} }} }}} \right)}}{{\mathop \sum \nolimits_{j = 1}^{n} \frac{{\phi \left( {x;x_{j} , \sigma^{2}_{{x_{j} }} } \right)}}{{p_{{x_{j} }} }}}},$$

where

6 $$p_{{x_{i} }} = \mathop \int \limits_{0}^{\infty } \phi \left( {x;x_{i} , \sigma^{2}_{{x_{i} }} } \right),$$

7 $$p_{{y_{i} }} = \mathop \int \limits_{0}^{\infty } \phi \left( {y;y_{i} , \sigma^{2}_{{y_{i} }} } \right).$$

Equation 5 represents the two-dimensional kernel density estimation for conditional probability, where the variation of *E*_rs_ depends on its prior state*. x*_*i*_ and *y*_*i*_ are the coordinates of a (*E*_rs*,N*+*1*_, *E*_rs*,N*_) data pair. Each $\phi \left( {x;x_{i} , \sigma^{2}_{{x_{i} }} } \right)$ and $\phi \left( {y;y_{i} , \sigma^{2}_{{y_{i} }} } \right)$ is a normal probability distribution function centred at a corresponding *x*_*i*_ and *y*_*i*_. Equation 6 and Eq. 7 are used to ensure that the probability distributions are properly normalised, where *p*_*xi*_ and *p*_*yi*_ represent the area under each normal distribution between zero and infinity and are therefore chosen to be non-negative. A full explanation of how to use this equation to form a stochastic model is found in previous works [44]. Once the stochastic model of *P(E*_rs*,N*+*1*_*|E*_rs*,N*_*)* is generated, its percentiles lines can be plotted (Fig. 3) and used as a simple look-up table to forecast potential a range of *E*_rs*,N*+*1*_ given *E*_rs*,N*_, with full details in [44]. Given a stochastic model for patient-specific, model-identified respiratory system elastance, it is possible to predict its range of potential changes over time.

### Decision support system protocol design

The designed protocol is named the Stochastic integrated VENT protocol (SiVENT). It is an extension of the VENT protocol in [45, 46]. The SiVENT protocol is divided into 3 phases.

#### Phase 1: identification of patient-specific information

The first phase identifies patient-specific *E*_rs_ and *R*_rs_ using integral-based parameter identification [69]. Patient weight can be collected from medical information on predicted body weight [70]. Identification is achieved using linear regression, per [69, 71]:

8 $$\dot{V}\left( t \right) = \frac{\text{d}V}{{\text{d}t}}.$$

Substituting Eq. 8 into Eq. 2:

9 $$P\left( t \right) = E_{rs} V\left( t \right) + R_{rs} \frac{\text{d}V}{{\text{d}t}} + \text{PEEP},$$

10 $$\mathop \int \limits_{{t_{0} }}^{{t_{i} }} P\text{d}t = E_{rs} \mathop \int \limits_{{t_{0} }}^{{t_{i} }} Vdt + R_{rs} \mathop \int \limits_{{t_{0} }}^{{t_{i} }} \frac{\text{d}V}{{\text{d}t}}\text{d}t + \mathop \int \limits_{{t_{0} }}^{{t_{i} }} \text{PEEP}\text{d}t,$$

11 $$\left[ {\begin{array}{*{20}c} {\begin{array}{*{20}c} {\mathop \int \limits_{{t_{0} }}^{{t_{1} }} Vdt} & {V_{1} - V_{0} } \\ {\mathop \int \limits_{{t_{0} }}^{{t_{2} }} V\text{d}t} & {V_{2} - V_{1} } \\ {\begin{array}{*{20}c} . \\ . \\ . \\ \end{array} } & {\begin{array}{*{20}c} . \\ . \\ . \\ \end{array} } \\ \end{array} } \\ {\begin{array}{*{20}c} {\mathop \int \limits_{{t_{0} }}^{{t_{N} }} V\text{d}t} & {V_{N} - V_{0} } \\ \end{array} } \\ \end{array} } \right]\left[ {\begin{array}{*{20}c} {E_{rs} } \\ {R_{rs} } \\ \end{array} } \right] = \left[ {\begin{array}{*{20}c} {\mathop \smallint \limits_{{t_{0} }}^{{t_{1} }} (P\text{d}t - \text{PEEP}) \text{d}t} \\ {\begin{array}{*{20}c} {\mathop \int \limits_{{t_{0} }}^{{t_{2} }} (P\text{d}t - \text{PEEP}) \text{d}t} \\ {\begin{array}{*{20}c} {\begin{array}{*{20}c} . \\ . \\ . \\ \end{array} } \\ {\mathop \int \limits_{{t_{0} }}^{{t_{N} }} (P\text{d}t - \text{PEEP}) \text{d}t} \\ \end{array} } \\ \end{array} } \\ \end{array} } \right].$$

Equation 10 is arranged into a system of linear equations to form Eq. 11 which can then be solved to find *E*_rs_ and *R*_rs*.*_ This is done by integrating both sides of Eq. 10 between the limits of *t*_*i*_ and *t*_*0*_. The symbol *t*_*i*_ is the initial data point of each fitting window, where *i* = 1, 2, 3,…*N*. Meanwhile, *t*_*0*_ refers to the very beginning of a breath, at which point present the first values of pressure and flow. Each linear equation in Eq. 11 is a different the cumulative integral of a unique fitting interval. Equation 11 now resembles the form of a matrix, which can be solved using MATLAB’s ‘*lsqnonneg*’ to identify non-negative values of *E*_rs_ and *R*_rs_.

Pressure–volume breath data are used to identify *E*_rs_ and *R*_rs_ values, and hence before identification can be done, it is important for the breath data to be processed and filtered using established criteria to remove data that do not qualify as a ‘true breath’. To mitigate small fluctuations in data, the following criteria are used to define a breath. These criteria can also be found in previous works [44]:

- Start of inspiration is defined as the first overall increase in flow (flow rate > 0.1 L/s) and pressure (pressure > (PEEP + 2 cmH_2_O)). Data are checked over the next 8 data points (0.16 s) to ensure constant positive flow.
- Start of expiration is defined as the first overall decrease in flow (flow rate < − 0.1 L/s). Data are checked over the next 8 data points to ensure constant negative flow.
- Peak Inspiratory volume reaches a significant value (peak inspiratory volume > 40 mL which is ~ 10% of typical tidal volume).
- Peak inspiratory pressure (PIP) is in the inspiratory phase and is of significant value (PIP > (PEEP + 1 cmH_2_O), where typical PIP is ~ PEEP + 10–14 cmH_2_O).
- Expiration is detected within 4.125 s of calculated onset of inspiration as defined above, matching the expected respiratory rate in this cohort.

As these criteria to remove fluctuations are generally lenient, additional criteria are added to further remove noise and asynchronous breathing cycles. Patients sometimes exhibit asynchronous events or patient effort during breathing. These asynchronous breaths do not accurately reflect the underlying patient-specific pulmonary mechanics as the pressure and flow waveforms are distorted [50, 51] and therefore are eliminated. These further criteria used to identify a ‘true breath’ are listed below:

- Median model-fit error for a breathing cycle > 15%.
- Model-based estimated *E*_rs_ ≤ 0.
- Model-based estimated *E*_rs_ outside 5th and 95th percentile of collected patient-specific data for that patient.

Model-fit for the first criterion is calculated using the median absolute percentage error (APE) between the model’s estimated airway inspiration pressure (*P*_sim_) and measured airway inspiration pressure (*P*_mea_) shown in Eq. 12. Note, *P*_sim_ is calculated using the identified model-based *E*_rs_. If *P*_mea_ deviates from *P*_sim_ too much, its APE will exceed the threshold, indicating too much noise or asynchrony. These breaths are thus not included in this study:

12 $$\text{APE} = \text{median}\left( {\left| {\frac{{P_{{\text{sim}_{i} }} - P_{{\text{mea}_{i} }} }}{{P_{{\text{sim}_{i} }} }}} \right|} \right) \times 100.$$

#### Phase 2: stochastic forecasting of *E*_rs_

**Fig. 4:**
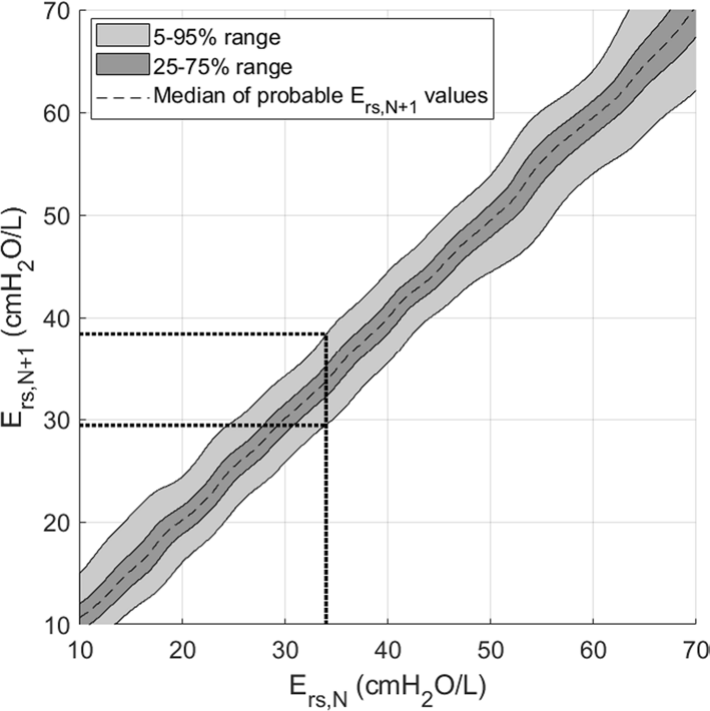
Obtaining the 5th and 95th percentile of *E*_rs*,N+1*_ using the stochastic model of *E*_rs_

Once, patient-specific parameters of *E*_rs_ and *R*_rs_ of the current interval, *N*, have been identified, the next phase will use the identified *E*_rs*,N*_ to forecast the potential range of *E*_rs_ in the subsequent future interval, *E*_rs*,N*+*1*_. This is done by feeding the current *E*_rs*,N*_ into the previously developed stochastic model of *E*_rs_ [44]. The output of the stochastic model will be a forecasted range of future *E*_rs*,N*+*1*_ as illustrated in Fig. 4. This protocol will make use of the forecasted 5–95th percentile prediction range of *E*_rs*,N*+*1*_ for use in the next phase of this protocol. More in-depth information regarding the stochastic model can be found in prior work [44].

#### Phase 3: VENT protocol

The predicted 5th and 95th *E*_rs_ values are used as inputs for the model-based VENT protocol [45, 46]. The VENT protocol consists of 4 stages: (1) virtual ventilation (*V*); (2) eliminate/estimate (*E*); (3) narrowing objectives (*N*); and (4) tabulation of data (*T*).

**Fig. 5:**
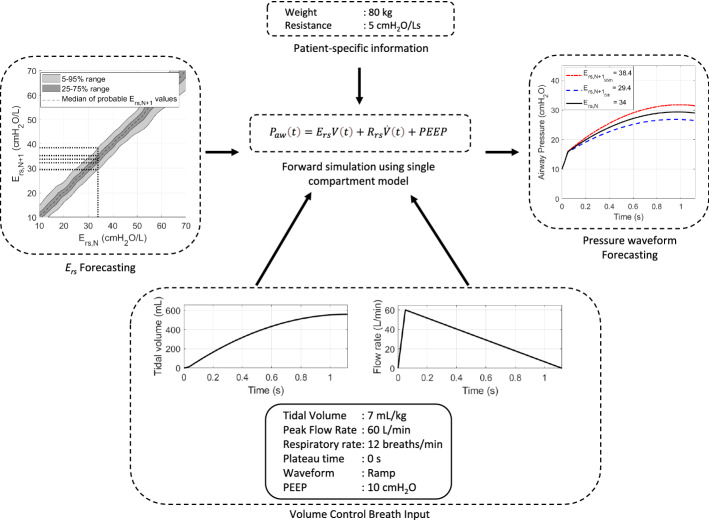
Visual illustration of forward simulation of pressure output waveform in VC ventilation provided a pre-determined combination of input settings and forecasted values of *E*_rs*,N+1*_

In the V-stage, all possible combinations of MV settings are forward simulated to forecast pressure outcomes. During volume control (VC) ventilation, adjustable parameter settings set by the clinician include PEEP (cmH_2_O), tidal volume, *V*_*T*_ (mL/kg), peak inspiratory flow rate, *V̇*_*MAX*_ (L/min), type of waveform (square or ramp), plateau time, *T*_*PLAT*_ (*s*), respiratory rate, RR (breaths/min) and I:E ratio [7, 8]. These settings form the volume, *V*, and flow, *V̇*, input waveform profiles along with PEEP in Eq. 2. Together with the patient-specific *R*_rs_ identified in Phase 1 and the forecast values of *E*_rs*,N*+*1*_ in Phase 2, the pressure output profile can be simulated using Eq. 2. This process is illustrated by example in Fig. 5. Once forecast pressure profiles are obtained, important VC ventilation outcomes such as peak airway pressure and plateau pressure for each variant of *E*_rs*,N*+*1*_ are recorded. This process is repeated for every possible combination of VC ventilation MV setting listed in Table 3. The result is a large collection of forecast data for every combination of possible MV settings.

**Table 3:** Resolution and range of VC ventilation parameter settings and total possible combinations

Setting	Resolution:range	Adjusted no. of combinations
Respiratory rate (breath/min)	1:6–35	30
Tidal volume (mL)	1:4–8	5
Peak inspiratory flow (L/min)	5:5–150	30
Plateau time (s)	0.1:0–2	21
Waveform	Square/ramp	2
Total number of possible mv setting combinations per patient = 1.89 × 10^5^		

In the E-stage, the collection of settings in the previous step is eliminated based on literature recommendations and clinical guidelines using the forecast pressure profiles. This filtering process eliminates setting combinations deemed unsafe based on well-accepted clinical guidelines. The recommended ranges of parameter settings are summarised in Table 4.

**Table 4:** Recommended parameter ranges based on literature

Outcome	Range	References
Respiratory rate, RR (breaths/min)	6–35	ARDSNet trial [10]
Tidal volume, *V*_*T*_ (mL/kg)	4–8	ARDSNet trial [10], State-of-the-Art review for Mechanical Ventilation in ARDS [72], Official Society of Critical Care Medicine Practice Guideline [7]
Positive end-expiratory pressure, PEEP (cmH_2_O)	Specified by clinician	ALVEOLI trial [4], LOVS trial [11], EXPRESS trial [12], Hogson staircase recruitment trial [73], Individualised PEEP trial [28], EPVent trial [74]
Peak pressure, *P*_MAX_ (cmH_2_O)	< 40	The basics of respiratory mechanics [75]
Plateau pressure, *P*_PLAT_ (cmH_2_O)	< 30	State-of-the-Art review for Mechanical Ventilation in ARDS [72], Official Society of Critical Care Medicine Practice Guideline [7]
I:E ratio	1:1–1:3	ARDSNet trial [10], ALVEOLI trial [4], LOVS trial [11], EXPRESS trial [12]

After eliminating harmful MV settings in the E-stage, the remaining settings can be further reduced by establishing clinically defined objectives in the N-stage. These objectives are set by clinicians based on individual and ICU objectives. Examples of narrowing objectives are minimising or maximising MV parameters, such as driving pressure, *∆P* (cmH_2_O), minute ventilation (L/min) or I:E ratio. As an example, clinicians can set the narrowing objective to reduce driving pressure, *∆P*, which is associated with reduced mortality [13, 31]. Hence, further MV setting combinations are eliminated based on these added narrowing objectives.

In the final T-stage, the remaining MV setting combinations are displayed in a table, where harmful combinations have been removed to meet the narrowing objectives set. An example is shown in Table 5. Note the forecast outcome displays the range forward simulated pressure outcomes using the 5th to 95th percentile of *E*_rs*,N*+*1*_ in the SiVENT protocol. The VENT protocol would display only a single value of forecast pressure outcomes for *E*_rs*,N*_, unable to account for intra-patient variability.

**Table 5:** Example of tabulation of data at the end of SiVENT protocol, showing remaining MV setting combinations recommended by protocol

No.	Input settings					5th to 95th percentile forecasted outcome			
	RR (breaths/min)	Waveform	*V*_*T*_ (mL/kg)	*V̇*_MAX_ (L/min)	*T*_PLAT_ (s)	*P*_MAX_ (cmH_2_O)	*P*_PLAT_ (cmH_2_O)	∆*P* (cmH_2_O)	I:E ratio
1	6	RAMP	4	10	0.1	12.1–13.6	11.9–13.4	4.4–5.9	1:1.8
2	6	RAMP	4	10	0.2	12.1–13.6	11.9–13.4	4.4–5.9	1:1.3
3	6	RAMP	4	10	0.3	12.1–13.6	11.9–13.4	4.4–5.9	1:1.1
4	6	RAMP	4	10	0.4	12.1–13.6	11.9–13.4	4.4–5.9	1:1.0
–	–	–	–	–	–	–	–	–	–
–	–	–	–	–	–	–	–	–	–
4666	35	SQUARE	4	60	0.5	36.6–40.0	11.9–13.4	4.4–5.9	1:1.3
4667	35	SQUARE	4	60	0.6	36.6–40.0	11.9–13.4	4.4–5.9	1:1.0

### Patient data and processing

This study uses retrospective airway pressure–flow data from 20 MV patients [76]. Ventilator data were recorded using CURESoft [77] connected to a Puritan Bennet PB980 ventilator (Covidien, Boulder, CO, USA). The study was approved by the IIUM research ethics committee (Ethics Approval Number IREC666). Patient-specific *E*_rs_ and *R*_rs_ are identified from the airway pressure–flow data [71, 78]. Further information on these patients and patient data processing can be found in [44]. All computation was done using MATLAB ver. R2020a (Natick, MA, USA).

### Model-based protocol performance evaluation

A case study is implemented to evaluate the performance of SiVENT and the potential benefit stochastic modelling adds to a model-based approach to MV care. The stochastic model free protocol omits step 2 of the SiVENT protocol, removing the stochastic forecasting component. The procedure for performance evaluation is defined:

1. Extract patient weight and identify patient-specific interval data, *E*_rs*,N*_ and *R*_rs*,N*_.
2. Extract clinically implemented interval PEEP, PEEP_*N*_.
3. Input patient profile (data obtained from steps 1 and 2) into VENT protocol and record the number of MV setting recommendations and percentage reduction from initial number of combinations.
4. Repeat step 3 for every interval up to 3 h of patient data.
5. Repeat steps 1 to 4 for each patient.
6. Repeat steps 1 to 5, replacing the VENT protocol with the SiVENT protocol in step 3.

The interval data refer to the median value of all data points within an interval, *N* where, *N* is set to *N* = 10 min in this study, but stochastic models can be developed for any clinically relevant interval. The narrowing objective implemented in this performance evaluation is to minimise driving pressure, *∆P*.

## Data Availability

Data are available upon request.

## Abbreviations

CT: Computed tomography
ICU: Intensive care unit
*E*_rs_: Respiratory elastance
*E*_rs__*,N*_: Respiratory elastance or interval *N*
*E*_rs__*,N*__+__*1*_: Respiratory elastance or interval *N*+*1*
MV: Mechanical ventilation
*P*_*aw*_: Airway pressure
*P*_*0*_: Offset pressure
PEEP: Positive end-expiratory pressure
*R*_rs_: Respiratory resistance
*R*_rs__*,N*_: Respiratory resistance of interval *N*
RR: Respiratory rate
*T*_PLAT_: Plateau time
*V*: Volume
*V̇*: Flow
*V*_*T*_: Tidal volume
*V̇*_MAX_: Peak inspiratory flow rate
VC: Volume control
*∆P*: Driving pressure
